# Complete mitochondrial genome of *Onchocerca lupi* (Nematoda, Onchocercidae)

**DOI:** 10.1080/23802359.2021.1960211

**Published:** 2021-08-05

**Authors:** Chandler C. Roe, Jennifer Urbanz, Lela Andrews, Guilherme G. Verocai, David M. Engelthaler, Crystal M. Hepp, Jason W. Sahl

**Affiliations:** a The Pathogen and Microbiome Institute, Northern Arizona University, Flagstaff, AZ, USA; b Translational Genomics Research Institute, Flagstaff, AZ, USA; c Eye Care for Animals, Scottsdale, AZ, USA; d Department of Biological Sciences, Northern Arizona University, Flagstaff, AZ, USA; e Department of Veterinary Pathobiology, College of Veterinary Medicine & Biomedical Sciences, Texas A&M University, College Station, TX, USA

**Keywords:** *O. lupi*, mitogenome, Bayesian phylogenetic analyses, Nematode, Onchocercosis, Filaria

## Abstract

*Onchocerca lupi,* Rodonaja 1967, is an emerging, zoonotic filarial nematode parasite that causes ocular disease in dogs, cats, wild canids, and humans. It is the causative agent of ocular onchocercosis in canines with increasing incidence in both North America and the Old World during the early twenty-first century. We report the complete mitochondrial genome of an *O. lupi* isolate from a dog from Arizona, southwestern USA, and its genetic differentiation from related *Onchocerca* species. The whole mitochondrial genome was obtained from whole genome sequencing of genomic DNA isolated from an adult worm. This mitogenome is 13,766 bp in size and contains 36 genes and a control region. This mitogenome provides a valuable resource for future studies involving epidemiological surveillance, population genetics, phylogeography, and comparative mitogenomics of this emerging pathogen and other parasitic nematodes.

Nematodes of the genus *Onchocerca* are found world-wide and infect a range of hosts such as humans, wild and domestic ungulates and carnivores, and comprise in excess of 30 valid species (Lefoulon et al. 2017). *Onchocerca lupi* is a tissue-dwelling zoonotic filarial parasite that causes ocular onchocercosis in dogs, cats, coyotes, wolves, and recently, humans (Roe et al. 2020). The putative biological vector of *O. lupi* are black flies (Diptera: Simuliidae), which transfer the infective third-stage larva to the mammal definitive host. Infection of the definitive host is usually associated with ocular disease, and may lead to blindness. Since 2010, there have been increasing reports of onchocercosis in domestic dogs and cats in North America and areas of Europe, North Africa and the Middle East (Labelle et al. 2011; Otranto et al. 2013; McLean et al. 2017), recent reports in coyotes (Roe et al. 2020), and zoonotic infections (Eberhard et al. 2013; Cantey et al. 2016). There is currently a knowledge gap regarding the genomics, population structure, and phylogeography of *O. lupi.* Previous research has utilized single mitochondrial genes (COI, ND5) for phylogenetic analyses providing low resolution relationships from global samples (Roe et al. 2020; Verocai et al. 2016); however, recent research of other zoonotic parasites identified phylogenetic relationships using whole mitochondrial sequencing that were indiscernible when examining short gene sequences (Kinkar et al. 2016; Laurimäe et al. 2018). Here, we report the complete mitochondrial genome of a single *O. lupi* nematode from the United States.

One adult nematode was collected by a local veterinarian from a privately-owned, infected dog from Flagstaff, AZ, USA (35°11′53.05″N, −111°39′4.57″W) in 2010. The specimen was pulverized by reciprocal shaking with steel beads at 30 Hz and DNA was extracted using a phenol-chloroform protocol (Doyle and Doyle 1990) modified with prolonged heated lysis and stored at −20 °C. Taxonomic identity was determined by Sanger sequencing using the previously published COI mitochondrial gene primers (Hassan et al. 2015). Genomic DNA was prepared for sequencing using previously published methods (Roe et al. 2016) and sequenced on an Illumina HiSeq 2500 platform (Illumina, San Diego, CA). An *O. lupi* specimen was deposited at the Pathogen and Microbiome Institute (https://in.nau.edu/pmi/, Roxanne Nottingham, Roxanne.nottingham@nau.edu) under voucher number OL-202101.

Due to the presence of both nuclear parasite as well as host DNA, mitochondrial reads were extracted bioinformatically through alignment to *Onchocerca volvulus* reference mitogenome (AF015193; 13,747bp); reads that mapped to the *O. volvulus* mitochondrial genome were separated using SAMtools v1.9 (Li et al. 2009), assembled using SPAdes (Bankevich et al. 2012) to an average depth of 303X, and examined for circularity using Circlator (Hunt et al. 2015). The entire circularized *O. lupi* mitogenome is 13,766 bases (GenBank accession MW266120). The mitogenome consists of 12 protein-coding genes (PCG), 22 transfer RNA (tRNA) genes, 2 ribosomal RNA (rRNA) genes, and 1 noncoding region (NCR). One DNA strand serves as the template strand for all genes. All PCGs are syntenic with the *O. volvulus* mitogenome; however, total synteny differs by the placement of one tRNA-Lys. The nucleotide distribution of the mitogenome was biased toward A + T (68%) which is similar to other reported filarial nematodes (Keddie et al. 1998).

Bayesian phylogenetic analyses of seven *Onchocerca* samples were implemented with the program ExaBayes v1.5.1 (Aberer et al. 2014) using the concatenated, shared SNP loci produced by the SNP pipeline NASP (Sahl et al. 2016), which spanned 12,259 shared positions (89%) across four *Onchocerca* species with the canine heartworm, *Dirofilaria immitis*, serving as the outgroup (Figure 1) . This analysis revealed a total of 2,260 SNPs; 852 SNPs were parsimony-informative positions revealing *O. lupi* as the most basal species within the genus *Onchocerca*, considering the limited number of species with characterized mitogenomes. We report the completed mitogenome of *O. lupi* by the next-generation sequencing and molecular phylogenetic placement within the genus *Onchocerca*. This mitogenome will provide a novel, strong foundation for further studies involving phylogenetic relationships among *Onchocerca* species and observations of the origin and range expansion of *O. lupi*.

**Figure 1.**
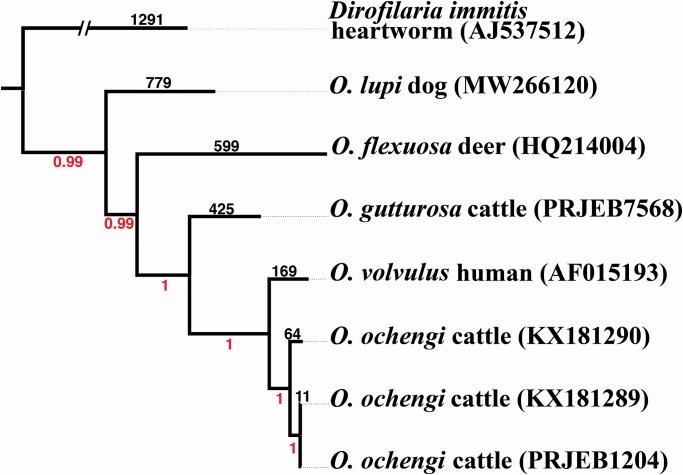
Bayesian phylogenetic relationships of *Onchocerca lupi* and 3 additional *Onchocerca* species based on concatenated SNPs that covered 89% total bases of the reference *O. lupi* mitogenome. Bayesian posterior probability values are shown below branches while SNP numbers are shown above corresponding branches. A“//” indicates a broken branch length that was shortened for visual purposes .

## Data Availability

The genome sequence data that support the findings of this study are openly available in GenBank of NCBI at https://www.ncbi.nlm.nih.gov/nuccore/MW266120.1 under the accession no. MW266120. The associated BioProject and Bio-Sample numbers are PRJNA733160 and SAMN19369283.
